# Modulation of Gene Expression in *Actinobacillus pleuropneumoniae* Exposed to Bronchoalveolar Fluid

**DOI:** 10.1371/journal.pone.0006139

**Published:** 2009-07-03

**Authors:** Abdul G. Lone, Vincent Deslandes, John H. E. Nash, Mario Jacques, Janet I. MacInnes

**Affiliations:** 1 Department of Pathobiology, Ontario Veterinary College, University of Guelph, Guelph, Ontario, Canada; 2 Groupe de Recherche sur les Maladies Infectieuses du Porc, Université de Montréal, St-Hyacinthe, Québec, Canada; 3 Centre de Recherche en Infectiologie Porcine, Université de Montréal, St-Hyacinthe, Québec, Canada; 4 Institute for Biological Sciences, National Research Council of Canada, Ottawa, Ontario, Canada; University of British Columbia, Canada

## Abstract

**Background:**

*Actinobacillus pleuropneumoniae*, the causative agent of porcine contagious pleuropneumonia, is an important pathogen of swine throughout the world. It must rapidly overcome the innate pulmonary immune defenses of the pig to cause disease. To better understand this process, the objective of this study was to identify genes that are differentially expressed in a medium that mimics the lung environment early in the infection process.

**Methods and Principal Findings:**

Since bronchoalveolar lavage fluid (BALF) contains innate immune and other components found in the lungs, we examined gene expression of a virulent serovar 1 strain of *A. pleuropneumoniae* after a 30 min exposure to BALF, using DNA microarrays and real-time PCR. The functional classes of genes found to be up-regulated most often in BALF were those encoding proteins involved in energy metabolism, especially anaerobic metabolism, and in cell envelope, DNA, and protein biosynthesis. Transcription of a number of known virulence genes including *apxIVA* and the *g*ene for SapF, a protein which is involved in resistance to antimicrobial peptides, was also up-regulated in BALF. Seventy-nine percent of the genes that were up-regulated in BALF encoded a known protein product, and of these, 44% had been reported to be either expressed *in vivo* and/or involved in virulence.

**Conclusions:**

The results of this study suggest that in early stages of infection, *A. pleuropneumoniae* may modulate expression of genes involved in anaerobic energy generation and in the synthesis of proteins involved in cell wall biogenesis, as well as established virulence factors. Given that many of these genes are thought to be expressed *in vivo* or involved in virulence, incubation in BALF appears, at least partially, to simulate *in vivo* conditions and may provide a useful medium for the discovery of novel vaccine or therapeutic targets.

## Introduction

*Actinobacillus pleuropneumoniae* is a species-specific swine pathogen that causes a necrotizing, fibrinohaemorrhagic pneumonia with pleurisy [1]. Depending upon the immune status of the animal, disease can range in severity from peracute to chronic [2], [3]. Although a protective immune response is usually acquired through the adaptive immune system following acute infection, vaccines offer only partial protection against this organism.

The lungs, which are the primary site of infection by *A. pleuropneumoniae*, have a large surface area that is directly in contact with the external environment. There are no published data for swine, but in the human lung, there is an average of 480 million alveoli [4] with an area of 120 to 140 m^2^ [5]. The lungs are protected by both innate and adaptive immune systems. Two major components are involved in the innate immune system: a cellular component comprised of leukocytes as well as airway and alveolar epithelial cells, and a humoral component which includes surfactant lipids and proteins, collectins, defensins, cathelicidins, lysozyme, and lactoferrin [6]. Most of these innate immune components reside in a thin layer of fluid lining the lung epithelial cell layer and some have been shown to directly kill bacteria such as *Escherichia coli* and *Pseudomonas aeruginosa* [7]–[9]. Inhaled particles, including pathogens, first encounter the resident immune system in this fluid layer, which forms the first line of defense [10], [11]. Little work has been done to examine bacterial gene expression in BALF, but in a recent study Schwab et al. [12] found that *Myobacterium tuberculosis* genes encoding proteins which enabled the organism to use surfactant lipids as a substrate and those for synthesis of phthiolocerol dimycocerosate (PDIM), a protective cell wall component, were up-regulated in the presence of a whole lung surfactant preparation.

*A. pleuropneumoniae* is capable of overcoming innate pulmonary immune mechanisms of the pig. It can rapidly multiply and spread in naive herds, with some pigs dying within 24 h of infection without showing any clinical signs. Several virulence factors have been described in *A. pleuropneumoniae* to explain its pathogenesis; factors for colonization, nutrient acquisition, immune evasion and tissue destruction have all been implicated in the disease process [13], [14]. Although some aspects of pathogenesis can be explained by the production of tissue-damaging RTX toxins and the ability of the pathogen to acquire nutrients such as iron in the host, factors involved in bacterial survival and rapid multiplication in the host are largely unknown.

To identify genes that may be involved in survival and pathogenesis of *A. pleuropneumoniae* in the host we used porcine bronchoalveolar fluid as a medium to simulate, in part, the lung environment. By analogy with other species, BALF collected from swine likely contains plasma proteins and proteins with unknown functions [15] as well as proteins with diverse functions including anti-oxidation, lipid-metabolism, and tissue repair and proliferation in addition to innate immune components and dissolved minerals. Because BALF contains components that perform diverse functions in the lungs, *A. pleuropneumoniae* gene expression in this fluid could mimic gene expression in the host. Therefore, the objective of this study was to identify *A. pleuropneumoniae* genes that are differentially expressed in BALF to better understand survival and pathogenesis of this important swine pathogen early in the disease process.

## Results and Discussion

### Differential gene expression in BALF

The survival of *A. pleuropneumoniae* CM5 was assessed in BALF before carrying out experiments to identify differentially expressed genes since this fluid contains many antibacterial substances [6], [16]. No significant decrease was observed in *A. pleuropneumoniae* CM5 cell numbers following incubation for 30 min in BALF, while 70% of the *E. coli* DH5α cells were killed at this time.

Genes that were differentially expressed by *A. pleuropneumoniae* after 30 min of incubation in BALF were identified with DNA microarrays by hybridization of Cy3-labeled cDNA from the BALF-incubated bacteria (target sample) and Cy5-labelled cDNA from the BHI-incubated bacteria (reference sample). One hundred and fifty-six genes were differentially expressed in BALF at a false discovery rate (FDR) of 1.07%; 52 genes were down-regulated while 104 were up-regulated. Forty-one (26%) of these genes encode hypothetical proteins (Table 1).

**Table 1:** Functional classes of differentially expressed genes.

Functional class	No. up-regulated	No. down-regulated	Total differentially expressed genes
Protein biosynthesis	16	1	17
Amino acid biosynthesis	0	9	9
Cofactor biosynthesis (heme and vitamins)	6	0	6
Nucleotide biosynthesis	0	3	3
Lipid biosynthesis	1	0	1
Cell envelope biosynthesis	11	0	11
Detoxification and toxin production	5	1	6
DNA metabolism	8	0	
Energy metabolism	22	4	26
Protein folding and stabilization	0	4	4
Transcriptional regulators	0	3	3
Secretion and trafficking	13	5	18
Transposon functions	0	3	3
Unclassified and unknowns	22	19	41
Total	104	52	157

Differential expression of selected genes representing various biological functional classes of interest was confirmed by real-time PCR analysis. Although fold change in gene expression measured by real-time PCR was generally higher, there was a good correlation between the two data sets, and no genes that were deemed up-regulated with the microarrays were demonstrated to be down-regulated by qRT-PCR, and vice-versa (Table 2). The reason why the three *nqr* genes tested appeared to be overestimated in the microarray analysis is not clear, but these slightly divergent results were not likely due to dynamic range or % G+C considerations.

**Table 2:** Verification of microarray data by real-time PCR.

Gene	Gene name	Fold change by real-time PCR	Fold change by microarray
*dmsA*	Anaerobic dimethyl sulfoxide reductase chain A precursor	17.90±6.52	5.74
*dmsB*	Anaerobic dimethyl sulfoxide reductase chain B	10.12±3.34	2.78
*nqrB*	Na^+^-translocating NADH quinone reductase subunit B	4.62±1.63	7.65
*nqrC*	Na^+^-translocating NADH quinone reductase subunit C	4.57±1.3	6.35
*nqrE*	Na^+^-translocating NADH quinone reductase subunit E	4.84±1.59	6.36
*napB*	Nitrate reductase cytochrome ctype subunit	11.61±3.94	4.69
*napF*	Ferredoxin-type protein NapF	15.94±5.35	6.42
*napD*	Putative NapD protein	18.59±7.25	3.93
*apxIVA*	RTX toxin protein	4.07±2.02	1.93
*dapA*	Dihydrodipicolinate synthase	0.09±0.02	0.20
*leuC*	3-isopropylmalate dehydratase large subunit 2	0.15±0.14	0.28
*ilvH*	Acetolactate synthase small subunit	0.13±0.17	0.27

The genes found to be most frequently up-regulated in BALF were those encoding proteins involved in energy metabolism and in cell envelope, DNA, and protein biosynthesis (Table S1). Genes encoding proteins for co-factor biosynthesis, toxin production and secretion and trafficking of ions and biomolecules were also up-regulated while genes encoding proteins involved in protein folding and stabilization, nucleotide biosynthesis, and mobile elements were down-regulated. Representative genes belonging to these functional classes are described below.

### Modulation of gene expression for enhanced protein synthesis and energy generation in BALF

Incubation of *A. pleuropneumoniae* CM5 in BALF for 30 min resulted in increased expression of genes encoding 30S and 50S ribosomal subunit proteins and tRNA modification enzymes (Table S1). Such up-regulation of ribosomal genes could play a role in synthesis of the proteins described below.

Genes encoding proteins involved in energy metabolism were also up-regulated in BALF, with some showing an increase of more than 6-fold. Most of these genes encoded enzymes involved in anaerobic respiration, including those that were part of the dimethyl sulfoxide reductase (*dms*) operon), periplasmic nitrate reductase (*nap*) operon, nitrite reductase (*nrf*) operon and a primary dehydrogenase, hydrogenase 2 (*hya*) operon (Table S1). Dimethyl sulfoxide (DMSO) reductase catalyzes the transfer of electrons to dimethyl sulfoxide and other substrates; the periplasmic-nitrate and nitrite reductases are involved in transfer of electrons to nitrate and nitrite respectively [17]. Hydrogenase 2, a primary dehydrogenase, uses the hydrogen produced by formate hydrogen lyase from formate as a substrate [18] for energy production [19], [20]. A putative formate transporter, *focA*, was also up-regulated in BALF.

Previous studies have shown that *A. pleuropneumoniae* up-regulates transcription of genes encoding enzymes involved in anaerobic metabolism in porcine lungs and lung washings [21]–[23]. *A. pleuropneumoniae* recovered from BALF following infection have increased expression of hydrogenase 2 [21], aspartate ammonia lyase (Asp) [24] and DMSO reductase [25], with DMSO reductase levels being elevated in cells recovered from both acute and chronic infections [22], [23].

The components present in BALF that could lead to up-regulation of anaerobic energy-metabolism genes in *A. pleuropneumoniae* are largely unknown; however, glutathione in the airway epithelium might be an activator of HlyX, which is the *A. pleuropneumoniae* equivalent of FNR in *E. coli* [26]. For example, it has been reported HlyX up-regulates DMSO reductase (*dms*) and aspartate ammonia lyase (*asp*), which breaks down aspartate to fumarate and ammonia. Fumarate is used as an electron acceptor under anaerobic conditions in *A. pleuropneumoniae* [27]–[30]. The fact that a significant change of expression of *hlyX* was not observed may be because differences in the level of expression of this gene tend to be small. Moreover, like *fnr*, regulation of the *hlyX* gene product is likely affected by a multitude of factors including protein stability, growth phase and nutrient availability [31], [32].

Up-regulation of the genes encoding the periplasmic nitrate (Nap) reductase in BALF suggests a role for nitrate metabolism in *A. pleuropneumoniae* energy production in the host. Nap uses nitrate as an electron acceptor. As nitrate has a higher redox potential than most other electron acceptors under anaerobic conditions [17], [33] it is a preferred electron acceptor. Nitrate is formed from nitric oxide in the animal and is present in various body fluids [34]–[37] where it can serve as a cue for the up-regulation of nitrate-responsive genes in *A. pleuropneumoniae*. Nap has a higher affinity for nitrate than membrane-bound nitrate reductase (Nar) [38], and it can be used for nitrate utilization in body fluids with low nitrate concentrations such as are found in the respiratory tract.

Nitrite reductase (Nrf) is another nitrate metabolism-related enzyme whose genes were up-regulated in BALF. This enzyme converts nitrite, a potential bacterial cell-damaging substance produced by nitrate reductase, to ammonia. Nrf can also convert nitric oxide to ammonium [39], thus inactivating a key defense molecule of the host.

Given that *A. pleuropneumoniae* is a host-associated pathogen which resides in oxygen-deprived environments in both the acute and carrier states of the disease, the major production of energy is likely through anaerobic metabolism. The absence of three main TCA-cycle enzymes (citrate synthase, aconitase and isocitrate dehydrogenase) in the genomes of serotype 3 and serotype 5 *A. pleuropneumoniae* again points to the importance of anaerobic metabolism in the survival of this organism [40]. In addition, many upper respiratory tract pathogens including *Haemophilus influenzae*, *Pseudomonas aeruginosa*, *Pasteurella multocida, Neisseria meningitidis*, carry genes for anaerobic energy generation, consistent with the notion that anaerobic metabolism might have an important role in the survival and virulence of bacterial pathogens in the respiratory tract.

Some of the genes encoding enzymes involved in anaerobic energy production in *A. pleuropneumoniae* have been shown to be essential for virulence. For example, knockout mutants of *hlyX* are unable to survive in lung epithelium, sequestered lungs or tonsils [29]; *dmsA* mutants are attenuated in acute disease [25]; and *asp* mutants cause less severe lung lesions than the wild type organism [24]. Similarly, in *Bordetella pertussis*, another respiratory tract pathogen, the FNR homolog, Btr [41] is essential for survival of this pathogen in mice [42].

The role of the nitrate-inducible energy metabolism genes, *nap* and *nrf*, is unknown in *A. pleuropneumoniae*. Nitrate metabolism has, however, been shown to be essential for the entry and replication of *Salmonella* Typhi in epithelial cells [43] and for the survival and virulence of *Mycobacterium bovis* in mice [44], [45].

In addition to the genes encoding enzymes of energy metabolism discussed above, the transcription of Na^+^ -translocating NADH-quinone reductase (NQR) was also enhanced in BALF (Table 3 and S1). NQR is a primary Na^+^ pump that translocates Na^+^ ions outside the cytoplasmic membrane to generate a sodium motive force, instead of a proton motive force, for energy production [46], [47]. The NQR enzyme is a complex of six subunits encoded by the *nqrABCDEF* operon [48], [49]; all six genes are present in all of the genomes of *A. pleuropneumoniae* reported to date. Another Na^+^-cycling gene, *nhaB* (an Na^+^/H^+^ antiporter), which, like NQR, could be involved in energy generation or in sodium homeostasis [46], was also up-regulated in BALF.

**Table 3:** BALF up-regulated virulence-associated genes reported in other studies.

Genes	Type of study	Reference no.
*bioD1*, *nhaB*, *apxIVA*, *rps*, *dmsA*, *hya*	SCOTS (acute infection; 7 days PI))	[22]
*nqrB*, *dnaG*, *rpsT*, *rplL*, *rho*, *secA*, *truD*, *nusA*, *atpD*, *sap*, *rps*, *rpl*	SCOTS (chronic infection; 21 days PI)	[23]
*dmsA*	Knockout mutation	[25]
*nqr*, *hemA*, *napB*, *atp*, *ccm*, *recR*, *tonB*, *galU*, *cpxC, gloB*	Signature tagged mutagenesis (24 h PI)	[51]
*sec*, *nusG*	*In vivo* expression technology (12 and 16 h PI)	[84]
*exbB2*, *atp*, *dnaK*	Signature tagged mutagenesis (20 h PI)	[85]
*apxIVA, malF, malG,* APL_0668 (predicted periplasmic lipoprotein involved in iron transport)	*In vivo* transcript profiling of *A. pleuropneumoniae* by microarray	Deslandes (personal communication)

Complete gene names are given in Table S1.

In previous studies, the *nqrB* [23] and *nhaB* [22] genes and the NqrA (AopA) protein [50], which are all involved in Na^+^-cycling, have been reported to be up-regulated in *A. pleuropneumoniae* when it is grown *in vivo*. The importance of NQR, the major Na^+^-cycling enzyme, in survival and pathogenesis of *A. pleuropneumoniae* is unknown. However, BHI containing 2-*n*-nonyl-4-hydroxyquinoline *N*-oxide (HQNO), an inhibitor of NQR, does not allow growth of *A. pleuropneumoniae* CM5, while *E. coli* DH5α grows well in media containing HQNO (unpublished data). Further, in signature tagged mutagenesis studies, the *nqrB* gene was found to be essential for persistence *A. pleuropneumoniae* in the host [51].

Although genome sequencing has revealed that many bacterial pathogens possess homologues of *nqr* and other primary and secondary sodium pumps [46], the role of Na^+^ -cycling in pathogenesis is largely unknown, except in *Vibrio cholerae*. In *V. cholerae*, mutation of *nqr* results in increased expression of *toxT*, a positive regulator of virulence factors including cholera toxin and toxin co-regulated pilus [52], [53]. NQR is best known for its involvement in energy transduction, cytoplasmic pH regulation and ion homeostasis in marine and halophilic bacteria [54], [55].

Other BALF-up-regulated *A. pleuropneumoniae* genes encoding enzymes of energy metabolism included the heme exporter gene (*ccmC*), ATP synthase epsilon chain *(atpC)*, deoxyribosephosphate aldolase (*deoC)* and 1-phosphofructokinase (*fruK*). The *ccmC* gene is a part of the *ccmABCDEFGH* operon which encodes proteins required for maturation of cytochrome C [56], an essential component of the electron-transfer chain [57]; whereas AtpC is a part of the F1 complex of ATP synthase [58]; DeoC cleaves deoxyribose 5-phosphate to acetaldehyde and glyceraldehyde 3-phosphate for central carbon metabolism [59]; and FruK regulates the flow of glucose through glycolysis [60]. Thus in BALF, *A. pleuropneumoniae* enhances transcription of the genes encoding both the central carbon metabolism and the energy transduction proteins.

The down-regulation of genes encoding TCA cycle related enzymes, phosphoenolpyruvate carboxylase (*pepC* and succinylCoA ligase (ADP forming) subunit alpha (*sucD*) genes (Table S1) again points to the importance of anaerobic metabolism in *A. pleuropneumoniae*. Phophoenolpyruvate carboxylase catalyses carboxylation of pyruvate to oxaloacetate and succinyCOA ligase catalyzes the nucleotide-dependent conversion of succinyl-CoA to succinate [61], [62]. The genes encoding putative haloacid dehalogenase like hydrolase *(pfhB) and* xylose isomerase (*xylA*) were also down-regulated, which could be because of the absence of the substrates for these enzymes in BALF or because alternate pathways are preferable in that environment. Haloacid dehalogenase catalyzes dehalogenation of L-2-haloalkanoic acids to form D-2-hydroxyalkanoic acids [63] and xylose isomerase converts xylose to xylulose [64]. The xylose transport system permease gene (*xylH*) was also down-regulated in BALF as were the mannitol (PTS system mannitol-specific EIICBA component, *mtlA*) and ribose (D-ribose binding periplasmic protein precursor, *rbsB*) transport systems (Table S1) consistent with the absence of manitol and ribose in BALF or the presence of a preferred substrate. The ferritin-like protein 2 encoding-gene, *ftnB*, which is involved in protection against oxidative damage to iron-sulfur-containing enzymes such as the tricarboxylic acid (TCA) enzyme aconitase [65] was also down-regulated in BALF. Since *A. pleuropneumoniae* lacks aconitase, the target of FtnB is not obvious. This result nevertheless suggests that *A. pleuropneumoniae* is not under oxidative stress in BALF.

### Modulation of gene expression for survival and virulence in BALF

Following incubation in BALF, *A. pleuropneumoniae* CM5 up-regulates genes required for cell wall synthesis, repair and recombination of DNA, and secretion and trafficking of ions and biomolecules (Table S1).

Several genes encoding cell wall biosynthesis proteins were up-regulated in BALF, including those required for synthesis of peptidoglycan, LPS and integral membrane proteins (Table S1). Up-regulated genes for peptidoglycan biosynthesis enzymes included phosphoglucosamine mutase (*mrsA*), alanine racemase (*alr*), and D-alanyl D-alanine carboxypeptidase fraction A (*dacA*). MrsA converts glucosamine-6-phosphate to glucosamine-1-phophate which finally yields UDP-N-acetyl glucosamine for both peptidoglycan and LPS biosynthesis [66]–[68] while Alr catalyses the isomerization of L-alanine into D-alanine which is essential in bacteria for peptidoglycan biosynthesis [66], [69], and DacA catalyzes transpeptidation between neighboring peptide chains of N-acetylmuramyl-N-acetylglucosyl polysaccharides to produce cross-links in the cell wall. DacA can also act as a carboxypeptidase to control the amount of cross-linking in peptidoglycan [70], [71].

A semi-rough LPS is present in *A. pleuropneumoniae* serotype 1 [72], and the BALF-up-regulated genes encoding LPS biosynthesis proteins included the tetraacyldisaccharide 4′kinase (LpxK) required for lipid-A biosynthesis, and a bifunctional protein (HldE) and UTPglucose-1-phosphate uridylyltransferase (GalU), required for LPS core biosynthesis. Genes encoding capsular export proteins, CpxA (ATP binding protein) and CpxC (capsule polysaccharide export inner membrane protein) were also up-regulated in BALF. While the genes encoding peptidoglycan and LPS biosynthesis proteins described above are assumed to be essential for the survival of *A. pleuropneumoniae*, a clear role for capsular polysaccharides in the virulence of the bacterium has been demonstrated [73], [74]. In addition to cell surface polysaccharides synthesis genes, the genes encoding the outer membrane protein OmpW (outer membrane protein W precursor) and a lipoprotein (outer membrane antigenic lipoprotein B precursor) were also up-regulated in BALF. The up-regulation of genes encoding proteins of cell wall biosynthesis may help the organism to overcome cell surface-damaging components present in BALF.

Transcription of genes encoding proteins involved in replication, and recombination and repair was enhanced in BALF. Genes encoding subunits of DNA polymerase III, various recombination proteins of the RecF machine, and an exonuclease (*uvrA of uvrABC*) were all up-regulated in BALF. Replication and recombination are two intertwined processes [75]; enhancement of transcription of genes involved in these two processes is consistent with active replication of *A. pleuropneumoniae* in BALF. On the other hand, *rec2*, encoding recombination protein2 and involved in transport of DNA across the cell envelope in competent bacteria [76], was down-regulated in BALF as were 3 genes predicted to have transposon functions. The fact that the expression of transposases is reported to be associated with starvation and other stressful conditions is again consistent with BALF being a favorable environment for *A. pleuropneumoniae* [77].

For survival in the host, bacteria require nutrients for biosynthesis of various biomolecules. In BALF, *A. pleuropneumoniae* increased transcription of genes encoding proteins required for transport of various nutrients. For example, complex-carbohydrate transport genes *malF* and *malG*, involved in maltose and maltodextrin transport, were up-regulated in BALF. Similarly, Group A *Streptococcus* enhances transcription of genes encoding proteins required for maltodextrin uptake in saliva and Δ*malE:malT* strains are attenuated in their growth and in their ability to catabolize α-glucans [78]. Genes for amino acid (9) and nucleotide (3) biosynthesis were down-regulated suggesting that some or all amino acids and nucleotides were either directly or indirectly, available in BALF. Consistent with this finding, amino acid transporters such as BrnQ (for branched chain amino acid) and SdaC (for serine transport) were up-regulated. In contrast, the product of the glycerol uptake facilitator gene, *glpF*, which allows transport of glycerol, erythritol, pentitols, and hexitol,was down-regulated, however, this gene is known to be down-regulated presence of glucose [79].

In BALF, *A. pleuropneumoniae* also increased transcription of genes encoding proteins required for transport of iron and potassium. Genes encoding the cell membrane transport proteins, ExbD and ExbD2, and FbpB (iron (III) ABC transporter, ATP-binding protein), which are involved in energy-coupled transport of the iron-containing compound, transferrin were up-regulated in BALF, as was *fieF*. The cation efflux pump, FieF, probably protects the bacterium from ferrous iron toxicity [80]. The gene encoding PtsN (PTS system, nitrogen regulatory IIA like protein) was also up-regulated in BALF. PtsN has recently been shown to regulate transport of K^+^ through its interaction with a K^+^ transporter in *E. coli* and could be involved in ion homeostasis needed for optimal survival of *A. pleuropneumoniae* in BALF [81].

Incubation in BALF also led to increased expression of *A. pleuropneumoniae* CM5 genes encoding toxin synthesis and antimicrobial-resistance compounds. The ApxIV RTX toxin is reported to be expressed only *in vivo* [82], [83]. Following exposure to BALF we have shown in vitro expression of *apxIVA* for the first time. ApxIVA is a homolog of FrpC in *Neisseria meningitidis*. FrpC is involved in tissue invasion of *N. meningitidis* [84]. The role, if any, of ApxIV in the pathogenesis of *A. pleuropneumoniae*, however, remains to be demonstrated.

The *sapF* gene is a part of the *sapABCDF* operon was up-regulated in BALF. It is involved in resistance to antimicrobial peptides in *Vibrio fischeri* [85], and in non-typable *Haemophilus influenzae* [86]. Also, *sapD* mutants of non-typable *H. influenzae* have been shown to be attenuated in a chinchilla model of otitis media [87]. *A. pleuropneumoniae* possesses a complete *sap* operon, which could have significant role in the survival of the pathogen in the host. Another detoxification molecule, glyoxylase II (*gloB*) is an enzyme involved in conversion of dicarbonyl compounds to less reactive hydroxy acids [88] was also up-regulated. It has been shown to be essential for survival of *A. pleuropneumoniae in vivo* [51] likely by protecting the organism against harmful dicarbonyl compounds present in the host. Expression of the *ostA* gene was also enhanced in *A. pleuropneumoniae* CM5 after incubation in BALF. The role for OstA in *A. pleuropneumoniae* is not known at this time, but in *Helicobacter pylori*, OstA confers protection against organic solvents and antibiotics [89], and in *E. coli*, it is essential for survival and has a direct role in membrane biogenesis and effects the lipid:protein ratio of the cell membrane [90]. In *N. meningitidis*, OstA is required for LPS biosynthesis [90].

Transcription of *secB*, which is a part of the Sec machinery, was also enhanced in BALF. The Sec machinery plays a key role in the translocation of proteins across, and integration of some proteins into, the cytoplasmic membrane of bacteria [91]. In *A. pleuropneumoniae*, *secA* and *secB*, another protein of the Sec machinery, have been shown to be expressed *in vivo* during both acute and chronic infection [23], [92], [and 93].

Several genes encoding transcriptional regulators, protein stabilization and folding and transposon functions were down-regulated in BALF. The precise role of these down-regulated transcriptional regulators is unknown in *A. pleuropneumoniae*, but FadR (a member of the GntR family of regulators) is an activator of unsaturated fatty acid acid synthesis in *E. coli*, although the authors do note that the FadR regulon in other gammaprotobacteria such as *Haemophilus influenzae* is much smaller [94]. Nevertheless, it is reasonable to assume that fatty acids would be freely available in BALF and their synthesis would not be required [95]. MerR transcriptional regulators have similar N-terminal helix-turn-helix DNA binding regions and C-terminal effector binding regions specific to the effector. Most of these regulators respond to oxidative stress and the presence of heavy metals and antibiotics in the medium [96]. The down-regulation of a MerR transcriptional regulator is consistent with the absence of stressors in the medium.

Similarly, the precise role of protein folding and stabilization proteins in *A. pleuropneumoniae* has not been reported, but in other systems, proteins such as HtpG and HtpX are usually up-regulated during stress such as nutrient deprivation and their down-regulation is consistent with BALF being a comparatively non-stressful environment [97], [98].

In the current study, genes encoding 22 “unclassified or unknown” proteins were up-regulated while genes encoding a further 19 “unclassified or unknown” proteins were down-regulated. This large set of gene encoding unknown proteins could have a significant role in the survival and pathogenesis of *A. pleuropneumoniae*. In the future, it may be possible to predict functions of unknown genes by using bioinformatic approaches such have been developed for analysis of human microarray data [99].

In summary, incubation in BALF appears to simulate *in vivo* conditions and may provide a useful medium for the discovery of novel vaccine or therapeutic targets. In this environment, *A. pleuropneumoniae* is actively involved in protein and cell envelope biosynthesis and in general, BALF appears to provide a comparatively favorable and nutrient replete environment. Although more than 40% of the genes that were up-regulated following a 30 min exposure to BALF had been reported in earlier in vivo studies (Table 3), we have described an additional 70 genes whose precise role in survival and virulence of *A. pleuropneumoniae* is unknown and merit further study.

## Materials and Methods

### Collectin and concentration of bronchoalveolar fluid (BALF)

BALF was obtained from ten specific pathogen free pigs, each weighing about 15 kg. The pigs were euthanized, and the lungs were lavaged in situ using a catheter passed through a bronchoscope to instill 100 ml of sterile PBS into the trachea. After ∼1 min, lung washings were collected and centrifuged to remove cell debris. The cell-free lavage was concentrated with a 5 kDa molecular weight cut off ultrafiltration device, Vivacell 70 (Vivascience Ltd., Stonehouse, Gloucestershire,UK). A total of about 100 ml of BALF was collected from each pig and concentrated to a final volume of 5 ml. Concentrated BALF from each pig was pooled and sterilized by passage through a 0.22 µm membrane filter. From collection to concentration, BALF was kept at 4°C; the concentrated BALF was stored at −80°C for long-term storage. Molecules less than 5 kDa in molecular weight were not concentrated by this method; nevertheless, the fluid still contained these substances in concentrations found before ultrafiltration and the concentrated BALF represents alveolar epithelial fluid better than unprocessed BALF. The procedure used for BALF collection received approval from the Animal Care Committee of the University of Guelph and was consistent with the Guidelines of the Canadian Council on Animal Care.

### Assessment of bacterial survival in BALF

Exponential growth phase cultures of *A. pleuropneumoniae* CM5 and *Escherichia coli* DH5α were incubated in 2 ml of concentrated BALF at 37°C. As a control, bacteria were also incubated in phosphate-buffered saline (PBS). A 50-µl aliquot was taken from each of the cultures after 15 and 30 min of incubation in BALF and PBS and plated onto brain heart infusion (BHI; Becton, Dickinson and Company, Sparks, MD, USA) agar supplemented with 0.01% (wt/vol) β-nicotinamide adenine dinucleotide (NAD). The number of colony-forming units (CFU) was counted after incubation overnight at 37°C. The number of bacteria surviving in BALF at each time point was expressed as the percent of number of bacteria surviving in PBS.

The data were analyzed using one-way analysis of variance (ANOVA); the means were compared using Tukey's method.

### Culture conditions for identification of differentially expressed genes in BALF

The virulent *A. pleuropneumoniae* serotype 1 strain CM5 was grown in BHI (Becton, Dickinson and Company) broth supplemented with 0.01% (wt/vol) NAD, at 37°C to an OD_600_ of 0.7 (approximately 10^7^ CFU/ml). The cell suspension was split into two equal parts and centrifuged at 10,000 x*g* for 1 min to pellet the cells. One pellet was suspended in pre-warmed concentrated BALF and the other in fresh pre-warmed BHI broth supplemented with NAD. The volume of BALF and BHI broth used to suspend the cell pellets was equal to that of the culture from which the pellets were obtained, so that the resulting cell suspension contained approximately 10^7^ CFU/ml. The cell suspensions were incubated with constant agitation at 37°C for 30 min and harvested by centrifugation for RNA extraction.

### RNA extraction

RNA was extracted using Trizol Reagent (Invitrogen, Carlsbad, CA, USA) according to the instructions of the manufacturer. RNA quantity and quality was determined using an RNA 6000 Nano LabChip read in a Bioanalyzer 2100 instrument (Agilent Technologies, Santa Clara, CA, USA). RNA was treated with Turbo DNA-free (Ambion, Austin, TX, USA) to remove traces of contaminating DNA. For hybridization in microarray experiments, RNA was extracted from 3 independent biological replicates.

### Labeling of cDNA and microarray hybridizations

cDNA synthesis was carried out as described previously [100]. Briefly, RNA (15 µg per reaction) from target (BALF-incubated bacteria) and reference (BHI-incubated bacteria) samples was used to synthesize cDNA in the presence of amino-allyl-dUTP (Sigma-Aldrich, St. Louis MO, US), random octamer primers (Biocorps, Montreal, QC, Canada), and SuperScript II transcriptase (Invitrogen, Carlsbad, CA, US). cDNAs were labeled indirectly with mono-functional NHS-ester Cy3 or Cy5 dye (GE Healthcare, Buckinghamshire, UK) and the efficiency of the labeling reactions was determined spectrophotometrically. RNA from three independent biological replicates was used in the labeling reaction. Four hybridizations, including the dye-swap experiment, were carried out between the target and the reference samples. The microarray data from this study were submitted to the Gene Expression Omnibus repository at NCBI and assigned accession number GEO: GSE13006.

### Microarray chip design

The AppChip2 microarray chip used in this study is an evolved version of the AppChip1 chip, and like its predecessor, was a part of the *A. pleuropneumoniae* 5b L20 genome sequencing project [101]. For construction of AppChip2, open-reading-frame (ORF) PCR fragments of 160-nucleotide length and above were spotted in duplicate on microarray slides. The spots represent 2033 ORFs, covering 95% of the total ORFs, from the complete genome sequence of the organism. Spotted sheared genomic DNA from *A. pleuropneumoniae* L20 and porcine DNA are used as controls (http://ibs-isb.nrc-cnrc.gc.ca/glycobiology/appchips_e.html). Other details concerning chip production are described elsewhere [102].

### Microarray data analysis

Microarray image and data analysis were carried out using the TM4 Suite [103] of software. Briefly, images were analyzed with Spotfinder v3.1.1. Background intensity was subtracted from the integrated intensity of each spot, and the spots that were less than one standard deviation above background intensity were eliminated, as were ones with total intensity less than 10,000. Replicate spots were analyzed subsequent to LOWESS (locally weighted linear regression) normalization of the data. Genes that were represented by good quality spots on a minimum of three replicate slides were considered for downstream analysis using SAM (significance analysis of microarray) to identify differentially expressed genes. A median false discovery rate (FDR = expected rate of falsely identified up- or down-regulated genes [104]) of 1.07% was used to generate a list of differentially expressed genes, which were classified into various functional classes using the JCVI Comprehensive Microbial Resource [105] tool.

### Quantitative real-time PCR

RNA capacity (the maximum RNA concentration that can be used without affecting efficiency of reverse transcription), optimum primer concentration (list of primers is given in Table S2), and gene dynamic ranges were determined before carrying out real-time PCR for relative quantification of target genes. Synthesis of template cDNA was carried out in a 20-µl reaction mixture containing 500 ng RNA, using High Capacity cDNA Reverse Transcription Kit (Applied Biosystems, Streetsville, ON, Canada). SYBR-Green-dye-based real-time PCR methodology was carried out using MicroAmp Optical 96- well plates (Applied Biosystems) in a StepOnePlus thermocycler (Applied Biosystems) for relative quantification of target genes. The 20-µl PCR reaction mixtures contained 10 µl of 2× Power SYBR Green PCR Master Mix (Applied Biosystems), 100 nM of forward and reverse primer, and 5 µl of template cDNA. The real-time PCR thermal profile included heat-activation of AmpliTaq Gold DNA Polymerase at 95°C for 10 min, and three-step 40-cycle PCR of denaturation at 95°C for 15 sec, primer annealing and extension at 60°C for 1 min.

The Comparative C_T_ (or ΔΔ C_T_) method [106] where ΔΔ C_T_ = (C_T, target_−C_T, *syp*_)_BALF_−(C_T, target_−C_T, *syp*_)_BHI_ was used to determine the relative gene expression of the target genes in BALF. As an endogenous control, the level of prolyl-tRNA-synthetase gene expression was used to normalize target gene expression levels, since this gene exhibited the least variation in expression across various conditions in both the microarray and real-time PCR experiments. Three independent biological replicates were tested in triplicates in the PCR experiments for the relative quantification of target genes.

## Supporting Information

Table S1 (0.26 MB DOC)

Table S2 (0.04 MB DOC)
