# Supplementary material for: Modulation of Gene Expression in Actinobacillus pleuropneumoniae Exposed to Bronchoalveolar Fluid
Source: PLoS One. 2009 Jul 3;4(7):e6139. doi: 10.1371/journal.pone.0006139 (PMC2700959; doi:10.1371/journal.pone.0006139)
Supplement: Table S1 — (0.26 MB DOC) [file pone.0006139.s001.doc]

#### Table S1. Differentially expressed genes of bronchoalveolar lavage fluid-exposed *A. pleuropneumoniae* grouped according to biological role

| **ORF No.** | **Gene** | **Predicted protein product** | | **Fold change** |
| --- | --- | --- | --- | --- |
| **Protein biosynthesis** | | | | |
| APL_1769 | *rpsQ* | ribosomal protein | | 4.27 |
| APL_0638 | *nusA* | transcription elongation protein NusA | | 4.10 |
| APL_0247 | *rho* | transcription termination factor Rho | | 3.06 |
| APL_1785 | *rplQ* | ribosomal protein | | 2.76 |
| APL_0042 | *rlmB* | 23S rRNA (guanosine-2'-O-)-methyltransferase | | 2.71 |
| APL_1558 | *rpsT* | ribosomal protein | | 2.26 |
| APL_0399 | *ksgA* | dimethyladenosine transferase | | 2.02 |
| APL_0352 | *metG* | methionyl tRNA synthetase | | 1.97 |
| APL_0601 | *rpsl* | ribosomal protein | | 1.94 |
| APL_0641 | *truB* | tRNA pseudouridine synthase B | | 1.89 |
| APL_0575 | *deaD* | coldshock DEAD box protein A like protein | | 1.88 |
| APL_1721 | *rplL* | ribosomal protein | | 1.87 |
| APL_1770 | *rplX* | ribosomal protein | | 1.74 |
| APL_1383 | *dnaX* | tRNA (guanineN(7)) methyltransferase | | 1.74 |
| APL_0365 | *hrpA* | ATP dependent RNA helicase | | 1.71 |
| APL_1228 | *infA* | translation initiation factor IF1 | | 1.67 |
| APL_1070 | *ftnB* | ferritin-like protein 2 | | -2.29 |
|  |  | Mean | | 2.08 |
|  |  |  | |  |
| **Amino acid biosynthesis** | | | | |
| APL_1230 | *serB* | phosphoserine phosphatase | | -1.61 |
| APL_1155 | *metE* | 5methyltetrahydropteroyltriglutamate homocysteine methyltransferase | | -1.72 |
| APL_2023 | *hisB* | histidine biosynthesis bifunctional protein HisB | | -1.77 |
| APL_0708 | *serC* | phosphoserine aminotransferase | | -2.11 |
| APL_0727 | *ilvI* | acetolactate synthase large subunit | | -2.14 |
| APL_2019 | *hisG* | ATP phosphoribosyl transferase (ATP-PRTase) (ATP-PRT) | | -2.58 |
| APL_0139 | *leuC* | 3-isopropylmalate dehydratase large subunit 2 | | -3.49 |
| APL_0728 | *ilvH* | acetolactate synthase small subunit | | -3.67 |
| APL_0899 | *dapA* | dihydrodipicolinate synthase | | -4.84 |
|  |  | Mean | | -2.66 |
|  |  |  | |  |
| **Protein folding and stabilization** | | | | |
| APL_1039 | *htpX* | putative protease HtpXl-like protein | | -2.37 |
| APL_1705 | APL_1705 | FKBP type peptidylprolyl cistrans isomerase | | -2.38 |
| APL_0987 | *htpG* | chaperone protein HtpG | | -2.44 |
| APL_1034 | *prlC* | oligopeptidase A | | -3.07 |
|  |  | Mean | | -2.57 |
|  | | | | |
| **Cofactor biosynthesis** | | | | |
| APL_1523 | *chuW* | coproporphyrinogen III oxidase | | 2.24 |
| APL_0963 | *hemN* | oxygen independent coproporphyrinogen III oxidase | | 1.92 |
| APL_0404 | *hemA* | glutamyl tRNA reductase (GluTR) | | 1.82 |
| APL_1988 | *hemB* | delta-aminolevulinic acid dehydratase | | 2.54 |
| APL_0614 | *bioD1* | dethiobiotin synthetase 1 | | 2.50 |
| APL_1485 | *pdxY* | pyridoxamine kinase | | 1.89 |
|  |  | Mean | | 2.15 |
|  |  |  | |  |
| **Lipid biosynthesis** | | | | |
| APL_1107 | *plsB* | glycerol-3-phosphate acyl transferase | | 1.72 |
|  |  |  | |  |
| **Nucleotide biosynthesis** | | | | |
| APL_2018 | *purC* | | phosphoribosylaminoimidazole succinocarboxamide synthase (SAICAR synthetase) | -2.41 |
| APL_1106 | *purT* | | phosphoribosylglycinamide formyl transferase 2 | -3.91 |
| APL_1172 | *purD* | | phosphoribosylamine glycine ligase | -8.22 |
|  |  | | Mean | -4.85 |
|  |  | |  |  |
| **Cell envelope** |  | |  |  |
| APL_1086 | *ompW* | | outer membrane protein W precursor | 6.20 |
| APL_1454 | *mrsA* | | phosphoglucosamine mutase | 3.40 |
| APL_0186 | APL _0186 | | ADP heptose:LPS heptosyl transferase I | 2.96 |
| APL_1930 | APL _1930 | | outer membrane antigenic lipoprotein B precursor | 2.41 |
| APL_0402 | *hldE* | | bifunctional protein HldE | 2.25 |
| APL_1596 | *dacA* | | D-alanyl D-alanine carboxypeptidase fraction A | 1.96 |
| APL_1585 | *cpxA* | | ATP binding protein | 1.96 |
| APL_0252 | *alr* | | alanine racemase | 1.90 |
| APL_0651 | *galU* | | UTP glucose-1-phosphate uridylyl transferase | 1.87 |
| APL_1583 | *cpxC* | | capsule polysaccharide export inner membrane protein | 1.65 |
| APL_1278 | *lpxK* | | tetraacyl disaccharide 4' kinase | 1.62 |
|  |  | | Mean | 2.56 |
|  |  | |  |  |
| **Detoxification and toxin production** | | | |  |
| APL_1379 | *ccp* | | cytochrome c peroxidase | 7.51 |
| APL_0998 | *apxIVA* | | RTX toxin protein | 1.93 |
| APL_0415 | *gloB* | | putative hydroxyacyl glutathione hydrolase | 1.86 |
| APL_1249 | *sapF* | | peptide transport system ATP binding protein SapF | 1.74 |
| APL_0962 | *ostA* | | organic solvent tolerance protein precursor | 1.60 |
| APL_0766 | *rec2* | | recombination protein 2 | -2.42 |
|  |  | | Mean | 2.04 |
|  |  | |  |  |
| **DNA metabolism** | | | | |
| APL_0074 | *recR* | | recombination protein RecR | 2.22 |
| APL_1602 | *mutS* | | DNA mismatch repair protein MutS | 2.18 |
| APL_1282 | *dnaQ* | | DNA polymerase III subunit epsilon | 2.15 |
| APL_0874 | *holA* | | DNA polymerase III subunit delta | 1.79 |
| APL_0545 | *recO* | | DNA repair protein RecO | 1.70 |
| APL_1474 | *dnaG* | | DNA primase | 1.62 |
| APL_0962 | *recQ* | | ATP dependent DNA helicase RecQ | 1.60 |
| APL_0782 | *uvrA* | | UvrABC system protein A | 1.49 |
|  |  | | Mean | 1.84 |
|  |  | |  |  |
| **Mobile elements** | | | |  |
| APL_0612 | APL _0612 | | putative transposase | -2.39 |
| APL_1057 | APL _1057 | | transposase | -2.74 |
| APL_1058 | APL_1058 | | transposase | -3.11 |
|  |  | | Mean | -2.75 |
|  |  | |  |  |
| **Energy metabolism** | | | |  |
| APL_1428 | *napG* | | ferredoxin type protein NapG like protein | 6.56 |
| APL_1431 | *napF* | | ferredoxin type protein NapF | 6.42 |
| APL_1426 | *napB* | | nitrate reductase cytochrome ctype subunit | 4.69 |
| APL_1430 | *napD* | | putative NapD protein | 3.93 |
| APL_1427 | *napH* | | ferredoxin type protein NapH like protein | 3.88 |
| APL_0101 | *nrfB* | | cytochrome c type protein NrfB precursor | 3.61 |
| APL_0151 | *nqrB* | | Na+-translocating NADH-quinone reductase subunit B | 7.65 |
| APL_0154 | *nqrE* | | Na+-translocating NADH-quinone reductase subunit E | 6.36 |
| APL_0152 | *nqrC* | | Na+-translocating NADH-quinone reductase subunit C | 6.35 |
| APL_0102 | *nrfC* | | nitrate reductase | 5.78 |
| APL_1674 | *dmsA* | | anaerobic dimethyl sulfoxide reductase chain A precursor | 5.74 |
| APL_1331 | *hyaA* | | hydrogenase2 small chain precursor | 5.63 |
| APL_0344 | *fruK* | | 1-phosphofructokinase | 4.70 |
| APL_1676 | *dms C* | | anaerobic dimethyl sulfoxide reductase chain C | 3.99 |
| APL_1675 | *dmsB* | | anaerobic dimethyl sulfoxide reductase chain B | 2.78 |
| APL_1334 | *hyaB* | | hydrogenase2 large chain precursor | 2.51 |
| APL_1645 | *atpc* | | ATP synthase epsilon chain | 2.38 |
| APL_1015 | *deoC* | | deoxyribose phosphate aldolase | 2.25 |
| APL_1037 | *focA* | | putative formate transporter | 2.12 |
| APL_0322 | *nhaB* | | Na+/H+ antiporter 2 | 2.03 |
| APL_1370 | *ccmC* | | heme exporter protein C | 1.79 |
| APL_0991 | *nrfD* | | nitrite reductase transmembrane protein | 1.64 |
| APL_1908 | *xylA* | | xylose isomerase | -2.07 |
| APL_0451 | *sucD* | | succinylCoA ligase [ADP forming] subunit alpha | -2.31 |
| APL_0132 | *pfhB1* | | putative haloacid dehalogenase like hydrolase | -3.46 |
| APL_0339 | *pepC* | | phosphoenolpyruvate carboxylase | -8.36 |
|  |  | | Mean | 2.95 |
|  |  | |  |  |
| **Secretion and trafficking** | | | | |
| APL_0077 | *exbD2* | | biopolymer transport protein ExbD2 | 8.21 |
| APL_1239 | *malG* | | maltose transport system permease protein | 6.08 |
| APL_0078 | *exbB2* | | biopolymer transport protein ExbB2 | 4.97 |
| APL_1238 | *malF* | | maltose transport system permease protein MalF | 4.38 |
| APL_1991 | *brnQ* | | branched chain amino acid transport system carrier protein BraB (branched chain amino acid uptake) | 3.80 |
| APL_0856 | *sdaC* | | serine transporter | 3.70 |
| APL_0276 | *frpB* | | iron regulated outer membrane protein B | 2.55 |
| APL_0717 | APL _0717 | | iron(III) ABC transporter, ATP binding protein | 2.38 |
| APL_1509 | *secB* | | protein export protein SecB | 2.36 |
| APL_0335 | *ptsN* | | PTS system, nitrogen regulatory IIA-like protein | 2.09 |
| APL_0604 | *fieF* | | cation efflux pump FieF | 1.80 |
| APL_0301 | *tolR* | | biopolymer transport protein TolR | 1.77 |
| APL_1569 | *exbD* | | biopolymer transport protein ExbD | 1.75 |
| APL_1911 | *xylH* | | xylose transport system permease protein | -1.65 |
| APL_1672 | *rbsB* | | D-ribose binding periplasmic protein precursor RbsB | -2.25 |
| APL_1258 | *pstB* | | phosphate import ATP binding protein PstB | -2.93 |
| APL_1630 | *mtlA* | | PTS system mannitol-specific EIICBA component | -3.37 |
| APL_0374 | *glpF* | | glycerol uptake facilitator protein | -7.49 |
|  |  | | Mean | 1.56 |
|  |  | |  |  |
| **Regulatory proteins** | | | | |
| APL_0932 | APL _0932 | | putative HTH-type transcriptional regulator | -1.80 |
| APL_0571 | *gntR* | | HTH-type transcriptional regulator | -3.85 |
| APL_1262 | APL_1262 | | transcriptional regulator MerR family, | -5.97 |
|  |  | | Mean | -3.87 |
|  |  | |  |  |
| **Unclassified and unknowns** | | | | |
| APL_0668 | APL _0668 | | hypothetical protein | 8.01 |
| APL_1115 | APL _1115 | | hypothetical protein | 3.21 |
| APL_0637 | APL _0637 | | hypothetical protein | 3.15 |
| APL_0707 | APL _0707 | | hypothetical protein | 3.09 |
| APL_1626 | APL _1626 | | hypothetical protein | 2.97 |
| APL_0029 | APL _0029 | | ABC transporter periplasmic protein | 2.80 |
| APL_1360 | APL _1360 | | hypothetical protein | 2.48 |
| APL_1220 | APL _1220 | | hypothetical protein | 2.33 |
| APL_1836 | APL _1836 | | hypothetical protein | 2.13 |
| APL_0363 | APL _0363 | | hypothetical protein | 2.02 |
| APL_0695 | APL _0695 | | hypothetical protein | 1.92 |
| APL_1221 | APL _1221 | | hypothetical protein | 1.72 |
| APL_0734 | APL _0734 | | hypothetical protein | 1.64 |
| APL_0762 | APL _0762 | | hypothetical protein | 1.64 |
| APL_1381 | APL _1381 | | hypothetical protein | 1.60 |
| APL_1920 | APL _1920 | | site-specific recombinase | 1.60 |
| APL_0334 | APL _0334 | | hypothetical protein | 1.59 |
| APL_1722 | APL _1722 | | hypothetical protein | 1.58 |
| APL_1722 | APL _1722 | | hypothetical protein | 1.58 |
| APL_0576 | *nlpI* | | lipoprotein NlpI-like precursor | 1.46 |
| APL_1930 | APL _1930 | | hypothetical protein | 2.41 |
| APL_1006 | APL _1006 | | hypothetical protein | 2.32 |
| APL_0885 | APL _0885 | | hypothetical protein | -1.56 |
| APL_1900 | APL _1900 | | hypothetical protein | -1.59 |
| APL_1046 | APL _1046 | | lysine exporter protein | -1.67 |
| APL_1187 | APL _1187 | | hypothetical protein | -1.73 |
| APL_0426 | APL _0426 | | hypothetical protein | -1.77 |
| APL_0815 | APL _0815 | | hypothetical protein | -1.77 |
| APL_1415 | APL _1415 | | hypothetical protein | -1.86 |
| APL_0926 | APL _0926 | | hypothetical protein | -1.91 |
| APL_1980 | APL _1980 | | hypothetical protein | -2.29 |
| APL_0999 | APL _0999 | | hypothetical protein | -2.30 |
| APL_1017 | APL _1017 | | hypothetical protein | -2.50 |
| APL_1629 | *mtlD* | | mannitol 1 phosphate 5 dehydrogenase | -2.71 |
| APL_1088 | APL _1088 | | hypothetical protein | -2.79 |
| APL_1491 | APL _1491 | | hypothetical protein | -2.82 |
| APL_1574 | APL _1574 | | hypothetical protein | -2.82 |
| APL_1495 | APL _1495 | | putative transcriptional regulator | -3.01 |
| APL_0137 | APL _0137 | | hypothetical protein | -3.73 |
| APL_1855 | APL _1855 | | hypothetical protein | -3.84 |
| APL_1588 | APL _1588 | | predicted TRAP transporter solute receptor | -7.93 |
|  |  | | Mean | 0.06 |
|  |  | |  |  |
