# Supplementary material for: Modulation of Gene Expression in Actinobacillus pleuropneumoniae Exposed to Bronchoalveolar Fluid
Source: PLoS One. 2009 Jul 3;4(7):e6139. doi: 10.1371/journal.pone.0006139 (PMC2700959; doi:10.1371/journal.pone.0006139)
Supplement: Table S2 — (0.04 MB DOC) [file pone.0006139.s002.doc]

#### Table S2. Oligonucleotide primers used in real-time PCR experiments

| **Gene** | **Forward primer** | **Reverse primer** |
| --- | --- | --- |
| *dmsA* | ATGTTGCCGGACAAGCACAAGATG | TCTCAATGGACAACGGCTACCACA |
| *dmsB* | AACAGGCATCGATTGCACCGTTAC | ACTTGGACGTGCGTGTTTATTGGC |
| *nqrB* | TCGGTGCGGCGATTATCGTCTTTA | AAGTTGAATACGGTTGCCGTTGCC |
| *nqrC* | ACCCGGCTGACGATAAAGCGAATA | CCACTTGGTTTACTTTGCCCGCTT |
| *nqrE* | GCGCACTTGTTGAAGGTGTGGATT | AAACGATACGCCACCGAAGATTGC |
| *napB* | GCGCATGGCAACCTAAACATTGGT | TACAGGCTTTGCAGTAGCGGAAAC |
| *napF* | ACAACCGTCTCCGCAACTTCTACA | TTGGCTACAACGGAAGAAGCATGG |
| *napD* | TCGGCTAAAGCAAGCTGTCTGTCA | TAGCGCAAGTGAAAGCGGACATTC |
| *apxIVA* | TTGGACTTCACCTGCAAACATGCC | CGGGCAAATATTCCAAAGCGCAGA |
| *dapA* | CAACCTGCAACGCCACTATTGGTT | TACGACAGGCGAATCGACCACATT |
| *leuC* | CTTTGCCGCGAACTTCCACTTTCA | TGCGTTTGGTATCGGTACATCGGA |
| *ilvH* | GAAAGTTTAACCGTTGCGCCGACT | ACGTTCAATATGCTCGGTAGGGCT |
| *syp* | AAGAAACGCCGAATGATGCACAGG | ACACCTCGATAGCACCACCTTTGT |
